# Prioritization of novel ADPKD drug candidates from disease-stage specific gene expression profiles

**DOI:** 10.1016/j.ebiom.2019.11.046

**Published:** 2019-12-24

**Authors:** Tareq B. Malas, Wouter N. Leonhard, Hester Bange, Zoraide Granchi, Kristina M. Hettne, Gerard J.P. Van Westen, Leo S. Price, Peter A.C. 't Hoen, Dorien J.M. Peters

**Affiliations:** a Department of Human Genetics, Leiden University Medical Center, Leiden, the Netherlands; b OcellO B.V., Leiden, the Netherlands; c GenomeScan B.V., Plesmanlaan 1/D, 2333 BZ Leiden, the Netherlands; d Drug Discovery and Safety, Leiden Academic Center for Drug Research, Einsteinweg 55, 2333 CC, Leiden, the Netherlands; e Center for Molecular and Biomolecular Informatics, Radboud Institute for Molecular Life Sciences, Radboud University Medical Center Nijmegen, Nijmegen, the Netherlands

**Keywords:** Drug repurposing, Autosomal dominant polycystic kidney disease, RNA-Sequencing, 3D cyst assay, Cheminformatics

## Abstract

**Background:**

Autosomal Dominant Polycystic Kidney Disease (ADPKD) is one of the most common causes of end-stage renal failure, caused by mutations in *PKD1* or *PKD2* genes. Tolvaptan, the only drug approved for ADPKD treatment, results in serious side-effects, warranting the need for novel drugs.

**Methods:**

In this study, we applied RNA-sequencing of *Pkd1cko* mice at different disease stages, and with/without drug treatment to identify genes involved in ADPKD progression that were further used to identify novel drug candidates for ADPKD. We followed an integrative computational approach using a combination of gene expression profiling, bioinformatics and cheminformatics data.

**Findings:**

We identified 1162 genes that had a normalized expression after treating the mice with drugs proven effective in preclinical models. Intersecting these genes with target affinity profiles for clinically-approved drugs in ChEMBL, resulted in the identification of 116 drugs targeting 29 proteins, of which several are previously linked to Polycystic Kidney Disease such as Rosiglitazone. Further testing the efficacy of six candidate drugs for inhibition of cyst swelling using a human 3D-cyst assay, revealed that three of the six had cyst-growth reducing effects with limited toxicity.

**Interpretation:**

Our data further establishes drug repurposing as a robust drug discovery method, with three promising drug candidates identified for ADPKD treatment (Meclofenamic Acid, Gamolenic Acid and Birinapant). Our strategy that combines multiple-omics data, can be extended for ADPKD and other diseases in the future.

**Funding:**

European Union's Seventh Framework Program, Dutch Technology Foundation Stichting Technische Wetenschappen and the Dutch Kidney Foundation.

**Research in context**

**Evidence before this study**

Autosomal Dominant Polycystic Kidney Disease (ADPKD) is a progressive kidney disease, with 50% of patients reaching end-stage kidney disease at the age of 55. Fluid-filled cysts that gradually replace normal kidney parenchyma, accompanied by massive fibrosis, are identified as the main cause of renal failure. Tolvaptan is currently the only approved drug for ADPKD treatment, but with serious side-effects (i.e. diuresis). Therefore, there is a need for drugs that specifically target the formation and growth of cysts, to slow down or halt disease progression.

**Added value of this study**

Using a novel approach that combines bio and chemo-informatics, we repurpose drugs for the treatment of ADPKD. We compared transcriptomic data of ADPKD mouse models at different disease stages, as well as before and after drug treatment, to identify genes that are involved in ADPKD progression. By screening the ChEMBL drug-protein interaction database, we prioritized a list of candidate drugs that target ADPKD progression-associated genes. Finally, we showed that three out of six selected candidate compounds exhibit cyst-growth reducing effects in vitro, without toxic effects.

**Implications of all the available evidence**

We have identified three novel compounds that could be further investigated and developed for the treatment of ADPKD, these are Meclofenamic Acid, Gamolenic Acid, and Birinapant. Furthermore, our approach is applicable to other diseases, provided that high quality transcriptomic/proteomics data is available for integration with large scale drug affinity and activity data.

Alt-text: Unlabelled box

## 1 Introduction

Drug repurposing, defined as the application of known drugs and compounds to treat new indications, is seen as a bypass for the long and expensive process of developing new drugs. Estimates show that drug repurposing can save more than 50% of the cost and time needed to develop new drugs [1]. In the past, accidental discovery, unintended side effects or obvious follow-on indications have led to new uses of such drugs [2]. Notable examples of drug repurposing include, Minoxidil (originally tested for hypertension; now indicated for hair loss) and Viagra (originally tested for angina; now indicated for erectile dysfunction and pulmonary hypertension). Current drug repurposing efforts span the spectrum from blind screening chemical libraries against specific cell lines [3] or against cellular organisms [4], to serial testing in animal models [5], and to data-driven computational methods [6]. The latter category explores the fact that a single molecule can act on several targets, making it valuable to indications where these targets are also relevant [7]. Gene expression profiles generated with expression microarrays or RNA-sequencing, have been used for the identification of druggable targets and pathways [8], [9], [10] and are suited for the identification of drug repurposing candidates under the assumption that diseases that share aberrant molecular processes may be targeted by the same drugs. However, gene expression profiles have mainly be used in isolation and integrative approaches where gene expression profiles are combined with other information are scarce.

Here we have undertaken a novel approach to repurpose drugs for the treatment of Autosomal Dominant Polycystic Kidney Disease (ADPKD). ADPKD is a genetic disease of the kidney, with a prevalence of 4 to 10/10,000, it is one of the most common causes of end-stage renal failure [11,12]. ADPKD is characterized by the gradual replacement of normal kidney parenchyma by fluid-filled cysts and fibrotic tissue with age, ultimately leading to end-stage renal disease in most patients. The main genes mutated in patients with ADPKD are the *PKD1* and *PKD2* genes [13]. ADPKD shows variable disease progression, with 50% of patients developing end-stage kidney disease by the age of 60. While advances have been made in slowing the progression of some other forms of chronic kidney disease, standard treatments have not reduced the need for renal replacement therapy in ADPKD [14,15]. Unfortunately, several experimental interventions have recently failed to show significant benefit in slowing the rate of functional decline [16], [17], [18], while the interventions with positive outcomes, including the approved drug Tolvaptan, reported modest effects [19,20].

The difficulty in identifying drugs for ADPKD treatment can be partially attributed to the lack of understanding of the functions of the *PKD1* and *PKD2*-gene products, and on how their inactivation leads to cyst development. Strategies are focused on therapies that can slow the rate of disease progression in PKD patients. The identification of more and better drugs would require a macro-level understanding of the key molecular pathways contributing to cyst initiation and growth in patients and animal models. Transcriptomics deep-sequencing of disease states was proven successful in identifying promising drug candidates in several examples [21,22].

By sequencing mild, moderate and advanced stages of ADPKD mouse models, we identified genes involved in ADPKD progression. To further validate these genes involvement in disease progression, we compared their expression to the expression profiles of drug-treated ADPKD mouse models and looked-for gene expression alterations that are normalized after drug treatment. These genes have been included in a drug repurposing analysis in which targets of drugs published in ChEMBL have been compared to our expression profiles. This resulted in the identification of several drugs that potentially can be repurposed for ADPKD. We validated several of these compounds in a 3D cyst culture assay and propose them as potential candidates for ADPKD treatment (Supplementary Figure 1).

## 2 Materials and methods

### 2.1 Animal models and drug treatments

#### 2.1.1 Mice used in the ADPKD progression analysis

The inducible kidney-specific *Pkd1*-deletion mouse model (tam-KspCad-CreER^T2^;*Pkd1^lox2-11;lox2-11^*, referred to as iKsp-*Pkd1^del^*) and tamoxifen treatments have previously been described [23]. In this study mutant mice are called *Pkd1cko* mice. RNA sequencing was done on kidneys from 5 adult Wild-type (Wt) mice and 24 *iKsp-Pkd1^del^* mice with tamoxifen-induced gene disruption at the age of 38 or approximately 90 days (Mutant). Four mice per group were sacrificed at 2wk, 3wk and 6wks after tamoxifen administration. Five mice were sacrificed at 11wk of age, 4 at 12wk of age and 3 mice at 15wk after tamoxifen administration (Supplementary Table 1, Supplementary Figure 2). In addition, a young PKD model was analyzed with tamoxifen treatment at postnatal age of 10 days, as previously described [24], and the kidneys were harvested at age of 4.7 weeks (*n* = 3). Blood sampling and blood urea measurements were performed using Reflotron technology (Kerkhof Medical Service) as described previously [25]. Only male mice were used.

#### 2.1.2 Ethics statement

All the animal experiments were evaluated and approved by the local animal experimental committee of the Leiden University Medical Center (LUMC) and the Commission Biotechnology in Animals of the Dutch Ministry of Agriculture.

#### 2.1.3 Drug treated mice

Rapamycin (Sirolimus), Curcumin and soluble activin receptor IIB Fc (sActRIIB-Fc) treated *Pkd1cko* mice and controls were previously published [23,24,26] (Supplementary Figure 2).

#### 2.1.4 Measurement of disease progression in ADPKD model

2KW/BW was used as measurement for disease severity and strongly correlated with the cystic index (Supplementary Figure 3).

### 2.2 Statistical analysis

#### 2.2.1 Processing of RNA sequencing samples

RNA sequencing was performed on the Illumina® HiSeq 2500. The Illumina® mRNA-Seq Sample Prep Kit was used to process the sample according the Illumina protocol "Preparing Samples for Sequencing of mRNA" (1,004,898 Rev. D). Briefly, mRNA was isolated from total RNA using the oligodT magnetic beads. After fragmentation of the mRNA, a cDNA synthesis was performed. This was used for ligation with the sequencing adapters and PCR amplification of the resulting product. The quality and yield after sample preparation were measured with a DNA 1000 Lab-on-a-Chip (Agilent Technologies). The size of the resulting products was consistent with the expected size distribution (a broad peak between 300–500 bp on a DNA 1000 chip). Clustering and DNA sequencing using the Illumina cBot and HiSeq 2500 was performed according to manufacturer's protocols. A concentration of 15.0 pM of DNA was used. Detailed run information per group is provided in Supplementary Table 1. HiSeq control software HCS v2.2.38 was used. Image analysis, base calling, and quality check was performed with the Illumina data analysis pipeline RTA v1.18.64 and Bcl2fastq v1.8.4. All samples had a quality score Q30 for more than 93.6% of reads. Resulting reads were aligned to the mouse reference genome version GRCm38 using Tophat v.2.0.12 with default parameters [27]. The only exception is the use of the no-coverage-search which does not perform an initial coverage search against the genome, thus reducing substantially the computational time. After alignment, HTSeq-count (Version 0.6.1) was used to estimate gene expression by counting reads that were mapped to the reference genome GRCm38 exons of each gene using the following options: -s (stranded) = no, -a (mapping quality) = 10, -m (mode) = intersection-nonempty, -i (identification) = gene_id -t (feature to count) = exon. Gene counts were transformed to Counts Per Million (cpm) values and then normalized using the TMM normalization method from the edgeR package (Robinson, McCarthy et al., 2010) (version 3.2) was used. Normalized genes were then used as an input for the Voom transformation method implemented in the *limma* package [28] in R 3.4.4. Genes with low expression values (cpm < 2 in more than 50% of the samples) were excluded from differential gene expression analysis. A linear-model was fit and differentially expressed genes were calculated across all samples involved in ADPKD progression and treated vs. untreated ADPKD samples. Raw data was deposited in ArrayExpress and given the following identifier E-MTAB-8086.

Validation datasets [15,29] were acquired from GEO (ID: GSE72554 and GSE7869) and further processed using *limma* for the identification of the differentially expressed genes in each of the different mice groups. For the data of Menezes et al., we compared the resultant lists of differentially expressed genes with the different clusters involved in ADPKD progression using the representation factor. The representation factor is the number of overlapping genes divided by the expected number of overlapping genes drawn from two independent groups. A representation factor > 1 indicates more overlap than expected of two independent groups, a representation factor < 1 indicates less overlap than expected, and a representation factor of 1 indicates that the two groups have the same overlap for independent groups of genes. For the data of Song et al., we combined the differentially expressed genes (P-value < 0.05, t-statistics) of the small and medium cysts and processed them using the method detailed in “Annotation of Gene Expression Profiles” sub-section.

#### 2.2.2 Principal component analysis (PCA)

Samples involved in ADPKD progression were selected and prepared for Principal Component Analysis (PCA). Briefly, the above noise level voom-transformed gene expression values were organized in a data matrix and given as an input for the *ir.pca* function in R. The loadings of the different principal components were plotted in a 2-dimensional plot using the *ggplot2* package in R.

#### 2.2.3 Gene expression clustering

Hierarchical clustering was applied on all differentially expressed genes resulting from the pairwise comparisons of all samples involved in ADPKD progression (*FDR < 0.005,* Supplementary Table 1*).* The *hclust* package in R was applied on the euclidean distance matrix calculated using the *dist* R function. Utilizing the *cutree* function implemented in R, the resultant clustering tree was cut into 15 clusters. For each cluster, all gene members were plotted. In addition, the average gene expression pattern was based on the averaged expression values at each time-point.

#### 2.2.4 Annotation of gene expression profiles

We annotated the resulting gene expression profiles using the GeneTrail2 v1.5 tool [30]. We ran the over-representation analysis against the Wikipathways database. We used all expressed genes above noise level as background and accepted enriched terms with *P-value* < 0.05.

#### 2.2.5 Drug targets acquisition and prioritization

All high quality data on the selected protein targets were acquired from ChEMBL release 22 [31]. High quality was defined as follows: data points with a ChEMBL confidence score of 9 (direct single protein target assigned), with a pChEMBL activity value, and having >= 30 compound measurements per protein. The pChEMBL value is the negative logarithm of activity in molar for curve fitted activity values such as Ki, IC50, EC50, AC50, XC50. Furthermore, only human proteins were considered. This led to a total of 990 protein targets (directly assigned targets), and 356,396 interactions with 240,433 compounds. *Mus musculus* gene identifiers were converted to the homologous *homo sapiens* identifiers using the BioMart tool on the Ensembl website [32], and cross-checked with the *Homo sapiens* drug targets. We prioritized the resulting drug targets from the ChEMBL database [33] through several filtering steps based on a couple of criteria. First the overlap between this set and the PKD progression genes was kept, a total of 168 protein targets with 54,698 annotated bioactivities, through 48,050 small molecules. Subsequently only drug targets that were annotated to small molecules that have been tested in phases 2, 3 or 4 of clinical trials were kept. This was aimed at keeping molecules that have passed phase 1, which is aimed at determining if a drug is safe for efficacy testing in phases 2 and 3, phases 4 represents approved and marketed drugs. Secondly, we filtered targets that have antineoplastic activity based on the Anatomical Therapeutic Chemical (ATC) Classification System [https://www.who.int/classifications/atcddd]. Thirdly, for all remaining drugs and targets, we investigated for each drug, its mode of action in relation to each of its remaining targets and compared this to the direction of deregulation in the PKD Progression. When a drug has a conflicting mode of action to what is needed to correct the target's expression in ADPKD, that drug received low priority. For example, if drugA is an agonist to an up-regulated target in PKD, drugA would be excluded (or receive low priority). We kept the drugs that did not have a known mode of action. Fourthly, for the remaining targets, we gave the highest priority to drug targets that were dysregulated in the early phases of the disease, followed by moderate phases and finally advanced phases.

### 2.3 3D cyst drug screening

The 3D cyst culture assay has been performed with *Pkd1*-KO mouse-inner medullary collecting duct (mIMCD3) cells (mIMRFNPKD 5E4) as described previously [34]. In short: mIMRFNPKD cells were mixed with Cyst-Gel (OcellO, Leiden, The Netherlands) to a final concentration of 150,000 cells/mL. 15μl of cell-gel mix was pipetted to 384-well plates (Greiner Clear, Greiner Bio-One B.V.) using a CyBio Felix 96/60 robotic liquid dispenser (Analyik Jena AG). After gel polymerization at 37 °C for 30 min, 33 μL culture medium was added to each well. Cells were grown in gel for 96 h, after which the cells were co-exposed with forskolin (Calbiochem) and one of the following molecules: Rapamycin (SelleckChem, S1039), Staurosporin (SelleckChem, S1421); Birinapant (Bioconnect, PK-CA577-2597–1), Gamma-Linolenic acid (Sanbio, 90,220–50), Eicosapentaenoic acid (Sanbio, 90,110–50), Meclofenamic Acid (Sanbio, 70,550–1), Zileuton (Sanbio, 10,006,967–10) and Indometacin (Sanbio, 70,270–1). Rapamycin and Staurosporin were used as cyst swelling inhibiting or toxic control respectively. All conditions were tested in quadruplicate. After 72 h, cultures were fixed with 4% Formaldehyde (Sigma Aldrich) and simultaneously permeabilized with 0.2% Triton-X100 (Sigma Aldrich) and stained with 0.25 M rhodamine-phalloidin (Sigma Aldrich) and 0.1% Hoechst 33,258 (Sigma Aldrich) in 1x PBS (Sigma Aldrich) for 12 h at 4C, protected from light. Imaging was done using Molecular Devices ImageXpress Micro XLS (Molecular Devices) with a 4x NIKON objective. For each well, 30 images in the Z- direction (50 μm apart) were made for both channels. Each image captures the whole well area. Image analysis for actin and nuclei was performed using Ominer analysis software (OcellO BV.) integrated in KNIME Analytics Platform (Konstanz, Germany, http://www.knime.org/). Further data analysis was also done in KNIME. The main read-out for efficacy, “cyst area”, was calculated per well as the average of the area in px of each object in every in-focus plain. This measurement was then normalized to positive (100%) and negative control (0%). The parameters used for toxicity; “nuclei area” and “nuclei roundness” were calculated in a similar fashion, “fraction apoptotic nuclei” was calculated as the amount of nuclei without actin signal relative to the total amount of nuclei, both as count-measurements. Graphs were made in Graphpad 6 (GraphPad Software, La Jolla, CA).

## 3 Results

### 3.1 Gene expression patterns associated with disease severity

**Fig. 1:**
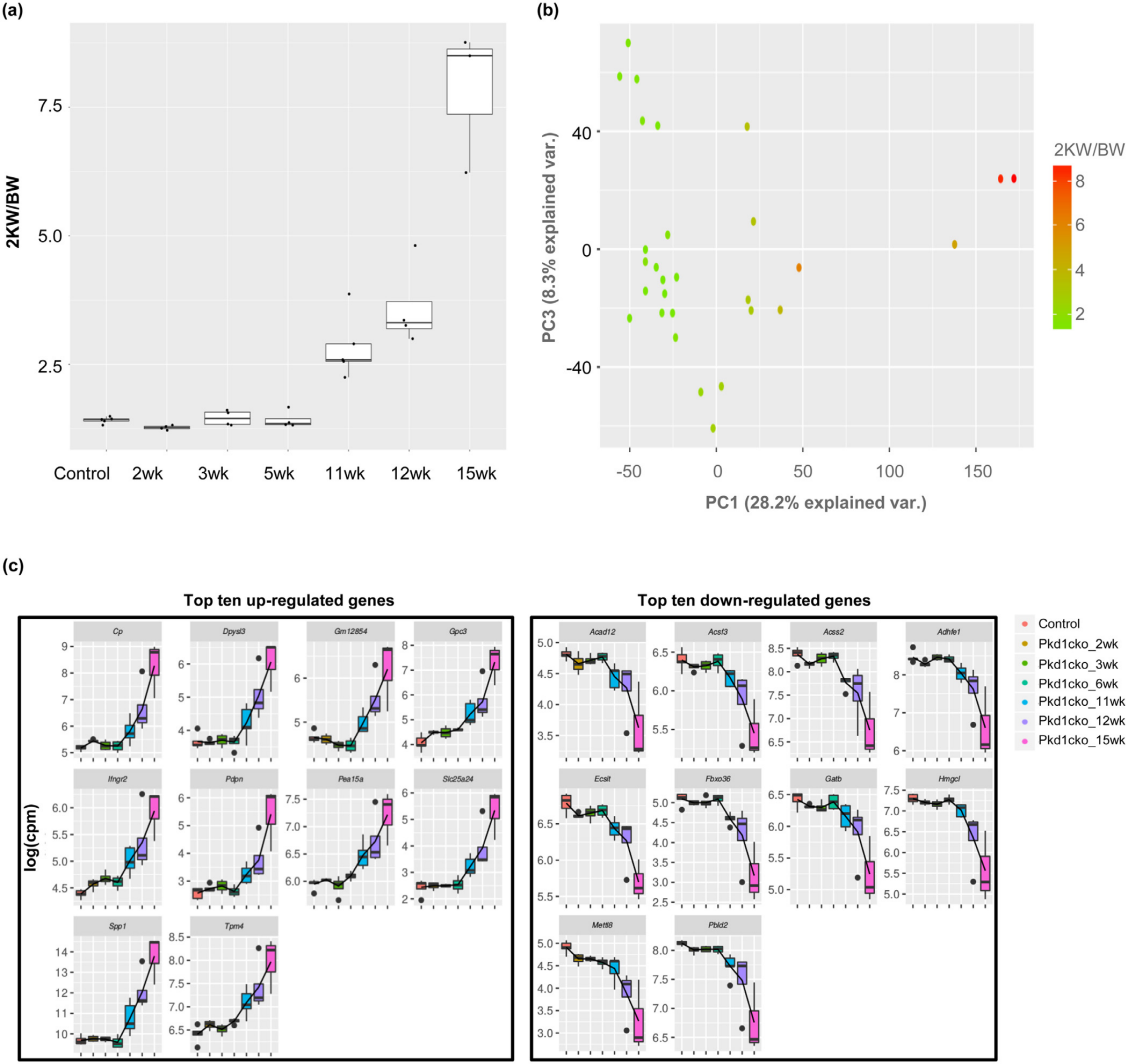
Kidneys taken out at various disease stages show differences in expression profiles. (a) Boxplot representation of the 2KW/BW values for groups of *Pkd1cko* mice representing different phases of ADPKD with increasing disease severity. (b) Results from principal component analysis of the *Pkd1cko* samples. Shown are the loadings of plot of pc1 (x-axis) and pc3 (y-axis) of all samples. In the panel the samples are colored based on their 2KW/BW value. (c) Boxplots of the top 10 most up-regulated (left part) and the 10 most down-regulated genes (right part) during disease progression, as extracted from the loadings of the genes on pc1. Expression data are given as log2 (counts per million).

To study the different phases of ADPKD progression, we have inactivated *Pkd1* at postnatal day 38 or 90 (adult phase) in the kidneys and harvested these animals at different time points after gene inactivation, resulting in five groups of mice with different disease stages. In these mice the largest group of cysts originate from the proximal tubules but cyst are also formed in distal tubules and collecting ducts [35]. *Pkd1cko* animals sacrificed after 2, 3 or 6 weeks after gene inactivation represent very early disease states. *Pkd1cko* animals sacrificed at 11 and 12 weeks after gene inactivation represent a moderate state of the disease and *Pkd1cko* animals sacrificed at 15 weeks after gene inactivation represent advanced disease. The kidney weight to body weight ratios (2KW/BW) of the five groups concurred with increasing disease severity in these samples (Fig. 1A, Supplementary Table 1).

We carried out RNA sequencing of the different groups of mice (Supplementary Figure 1). RNA was extracted from the five *Pkd1cko* and wild-type (WT) groups and cDNA sequenced on the Illumina 2500 Hiseq platform. Applying principal component analysis (PCA) on the gene expression profiles of these samples and plotting the first components revealed that most of the variance between samples could be attributed to differences in disease severity (principal component-1 (pc1), explaining 28% of the total variance, Fig. 1B). Extracting the 20 most influential genes in component-1 and plotting their expression in all disease progressing samples showed that these genes strongly correlated with disease progression (average Spearman's rank correlation coefficient = 0.7; Fig. 1C). Components-2 and 3 explained 26.7% and 8.3% of the variance respectively, where component 3 may reflect the variation between different mice.

### 3.2 Expression patterns associated with ADPKD progression

**Fig. 2:**
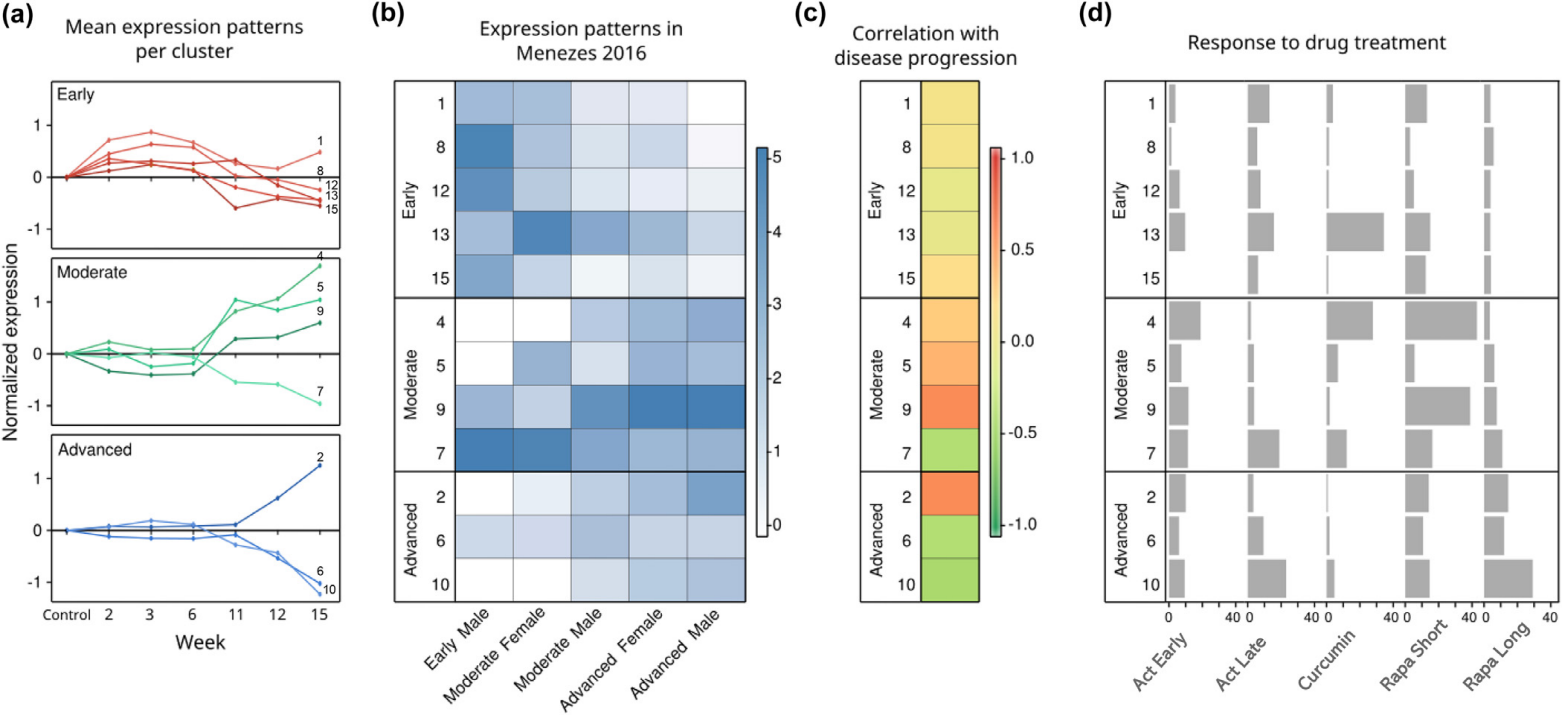
12 distinct expression patterns are associated with PKD progression. (a) The different expression patterns observed in *Pkd1cko* mice representing the progression of ADPKD towards end-stage renal disease (weeks after tamoxifen induction). For each cluster, mean log-transformed gene expression levels relative to the control mice that did not receive tamoxifen are plotted. The top panel represents the early dysregulated clusters, the middle panel represents the clusters dysregulated in the moderate to advanced stage and the bottom panel the clusters associated with the advanced stage of the disease. (b) Replication of expression profiles in an independent study. For each cluster, a representation factor reflecting the gene overlap of each cluster with the expression signatures from the five different mouse groups defined in the study by Menezes et al. [15] is given in a color representation. A representation score > 1 reflects enrichment. (c) Correlation of gene expression with disease progression. For each cluster, the average Spearman's correlation coefficient between the expression values of the genes in a cluster and the 2KW/BW ratio was calculated. Green represents a negative correlation while red reflects a positive correlation. Clusters that were dysregulated in an early stage have the lowest correlation with the 2KW/BW increase, suggesting they follow a different trend in disease progression. (d) Association of cluster with drug response. A bar chart representation for each cluster showing the proportion of the 2731 genes that were also affected by one of the drug treatments: sActRIIB-Fc early (Act Early) and late (Act Late), curcumin, rapamycin short (Rapa Short) and long (Rapa Long). The x-axis represents the % of genes that were significanltly dysregulated (P < 0.05) due to the drug treatments per cluster per drug treatment.

To gain fine-grained insights into the different patterns of gene expression during disease progression, we applied hierarchical clustering on the 2731 differentially expressed genes (*FDR < 0.005)* discriminating the groups of mice in different states of disease progression*.* In the first round of clustering we grouped the 2731 differentially expressed genes based on their expression patterns across the different disease progression stages into 15 clusters. This resulted in 12 gene clusters with distinct and coherent expression profiles, ranging in size from 32 to 367 genes. Additionally, three clusters contained genes that showed an aberrant pattern in just one of the samples. These clusters (cluster 3, 11 and 14) were removed from further analysis (Fig. 2A, Supplementary Table 2). The remaining 12 clusters were characterized by their gene expression patterns. For example, cluster 1 shows up-regulation in the early pre-cystic phases of the disease, particularly at 2wk, 3wk and 6wks after gene inactivation. Cluster 4 on the other hand includes genes that are up-regulated in the moderate phase of the disease starting from 11 weeks of gene inactivation. Cluster 10 is an example of a cluster that contains genes that are down-regulated in the advanced stages of the disease, at 12–15 weeks after gene inactivation. As we are interested in the three distinct phases of the disease (i.e. early, moderate or advanced), we further grouped the 12 clusters into 3 groups, where each of the new groups represents one of the three distinct phases, with genes up- or down- regulated particularly in early (*n* = 5 clusters), moderate (*n* = 4 clusters) or late (*n* = 3 clusters) phases of disease (Fig. 2A).

### 3.3 The expression patterns can be replicated in an independent study

Menezes et al. recently published a study of a different *Pkd1* knockout mouse model for ADPKD [15]. They included mouse samples at different disease stages namely, pre-cystic, cystic and severely cystic. We tested the statistical enrichment, using the representation factor (RF) method, of the genes in each of our three disease-stage groups were compared to the genes that are differentially expressed in pre-cystic, cystic and severely-cystic male mice of Menezes et al. As expected, the early dysregulated group demonstrated the strongest overlap with the pre-cystic groups in Menezes et al. study (Fig. 2B). Likewise, the moderate stage group showed greater overlap in the cystic and severely cystic groups. Similar patterns were observed in the advanced gene group, which was most consistent with the cystic and severely cystic groups (Fig. 2B). The strong overlap observed across disease stages was more evident in the up-regulated clusters compared to the down-regulated clusters. Taken together, these results reflect strong reproducibility of the expression patterns in an independent study. Since 2KW/BW is an accepted measurement of ADPKD disease stage and progression, we correlated the expression values in the 12 distinct clusters with 2KW/BW. The spearman coefficient plotted in Fig. 2C showed strong correlation of moderate and advanced stage clusters with 2KW/BW, while the early phase clusters had a weak correlation with 2KW/BW. This is expected, because the early ADPKD samples have 2KW/BW similar to that of the wild types.

### 3.4 Biological functions and pathways associated with ADPKD progression

**Fig. 3:**
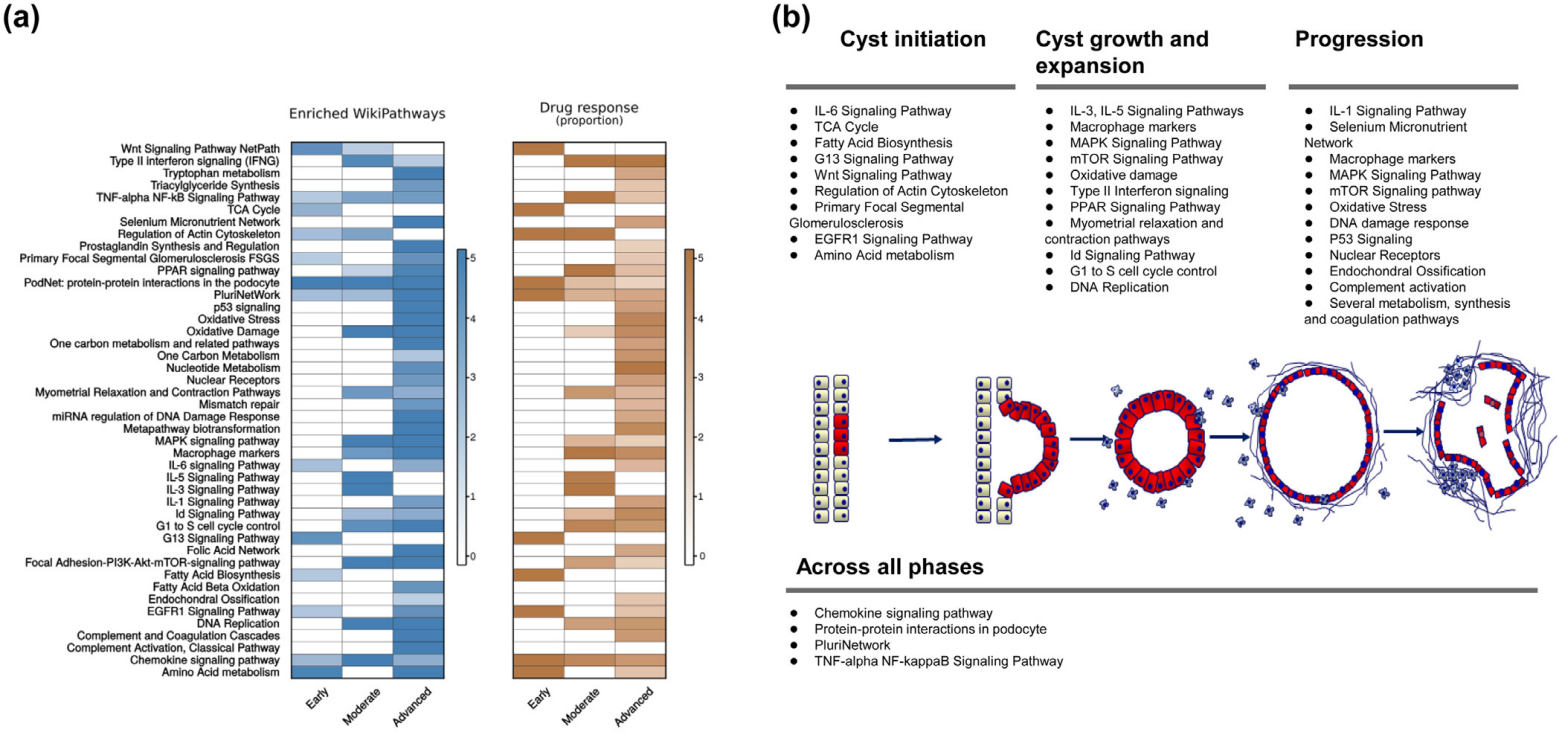
Pathways associated with disease progression and drug response. (a) A heatmap representation of the molecular pathways significantly enriched in the different stages of PKD (left). For each cluster category from Fig. 2A, the significantly enriched Wikipathways were obtained (*FDR < 0.05)* and plotted in the heatmap. Color scale reflects the representation factor in the different phases of ADPKD (Early, Moderate and Advanced). On the right, a heatmap representation of the pathways that are enriched with significantly dysregulated genes after drug treatment. Enrichment was established based on the representation (RF) factor calculation, where pathways that had RF >= 1 are considered significant. (b) A schematic representation of the different pathways involved in ADPKD's progression. Pathways are selected based on their Wikipathways significance (*FDR)* across the different disease stages in part A.

To understand the biological functions involved in ADPKD progression, we looked for the over-represented pathways in each of the three disease phases, early, moderate and advanced. For each disease phase, we combined the genes of the clusters that belonged to that phase and used GeneTrail2 v1.5 Wikipathways database to annotate them (*FDR* *<* *0.05*). Terms enriched (*FDR* < *0.05*) in any of the three disease phases are shown in Fig. 3A and provided as Supplementary Table 3. Hierarchical clustering was used to distinguish pathways that were specifically enriched in the early, moderate or late phases of the disease, and the pathways that were dysregulated across all phases (Fig. 3B). Interestingly, even at the pre-cystic phases we observed dysregulation in metabolism in the form of dysregulated TCA cycle and fatty acid biosynthesis, as well as Wnt signaling. Additionally, we observed dysregulation in G13 signaling pathway that is involved in cytoskeletal remodeling in cells and is essential for receptor tyrosine kinase-induced migration of fibroblast and endothelial cells. In the moderate and advanced phases of the disease, proliferation-related and inflammation-related pathways were dominant. The oxidative stress pathway, p53 and DNA mismatch-repair pathways were clearly visible in the advanced phase, along with alterations in metabolism. TNFα and chemokine signaling were active during all phases, from pre-cystic to advanced PKD. Using the work of Song et al. 2009 as a reference for human ADPKD, we confirmed the dysregulation of several of the aforementioned pathways in PKD patients. These include TCA cycle alterations, aberrant metabolism, active cytoskeleton remodeling and inflammation (Supplementary Table 3D).

### 3.5 Further selection of the ADPKD progression genes by evaluating response to therapy

We have previously shown that treating *Pkd1cko* P40 mice with Rapamycin and Curcumin and *Pkd1cko* P10 mice with soluble activin receptor IIB Fc (sActRIIB-Fc) significantly reduced kidney size and slowed the progression of ADPKD in mice [23,24,26] (Table 1). Here, we sequenced the RNA of the kidneys of these drug-treated mice using Illumina 2500 Hiseq platform and identified the differentially expressed genes (DEGs) between the treated and untreated samples. The curcumin treated samples are *Pkd1cko* P40 mice harvested at 11 weeks of age after gene inactivation; the same mouse model was treated with Rapamycin and harvested at two time-points, 12 weeks and 15 weeks after gene inactivation (Supplementary Figure 2). The soluble activin receptor-Fc fusion (sActRIIB-Fc) treatment was given to *Pkd1cko* P10 mice at two different time-points after tamoxifen treatment, starting at 0.3 weeks for the early-treated samples and at 2.1 weeks for the late treated mice (Supplementary Figure 2). Both groups were harvested at 4.1 weeks of age.

**Table 1:** The results of the RNA-Sequencing results of the drug treated samples (Curcumin, Rapamycin and sActRIIB-Fc). Significant genes (P-value < 0.05, t-statistics) were identified based on the comparison of the drug treated samples to the non-treated control (see methods for details) .

PubChem CID	Drug name and drug treatment (*Supplementary figure 2*)	No. of genes significant genes (*P-value < 0.05, t-statistics)*	Normalized no. of genes compared to PKD progression^a^	No. of genes found in PKD progression clusters
969,516	Curcumin	8030	840	503
5,284,616	Rapamycin Short	1600	840	441
5,284,616	Rapamycin Long	1250	840	322
NA	sActRIIB-Fc late-short treatment	840	840	270
NA	sActRIIB-Fc early-long treatment	4200	840	365

a Number is based on the lowest maximum of significant genes. This belongs to sActRIIB-Fc Late-Short treatment.

To balance the analysis between the different treatment groups, we took equally sized lists of the most differentially expressed genes (sorted on *P-value*). The size of the gene list was based on the treatment group with the lowest number DEGs (i.e. sActRIIB-Fc treatment), which is equal to 840 genes (P-value < 0.05 t-statistics, Table 1). For 1162 out of the 2731 genes that we identified to be involved in ADPKD progression, the expression was normalized after at least one drug treatment, i.e. upregulated genes were not or less increased after treatment, or downregulated were not or less decreased after treatment (Supplementary Table 2). Since the drug treatments were effective in slowing disease progression, these genes reflect the healthier state of the kidneys upon drug treatment. By focusing on genes that respond to therapy we strengthen the involvement of the genes in disease progression and as potential target to identify novel drugs to treat ADPKD (Figure-2D Fig. 2D). Table 1 summarizes the number of genes differentially expressed in the treated samples and involved in ADPKD progression.

### 3.6 Identifying drug targets from the genes associated with ADPKD progression

**Fig. 4:**
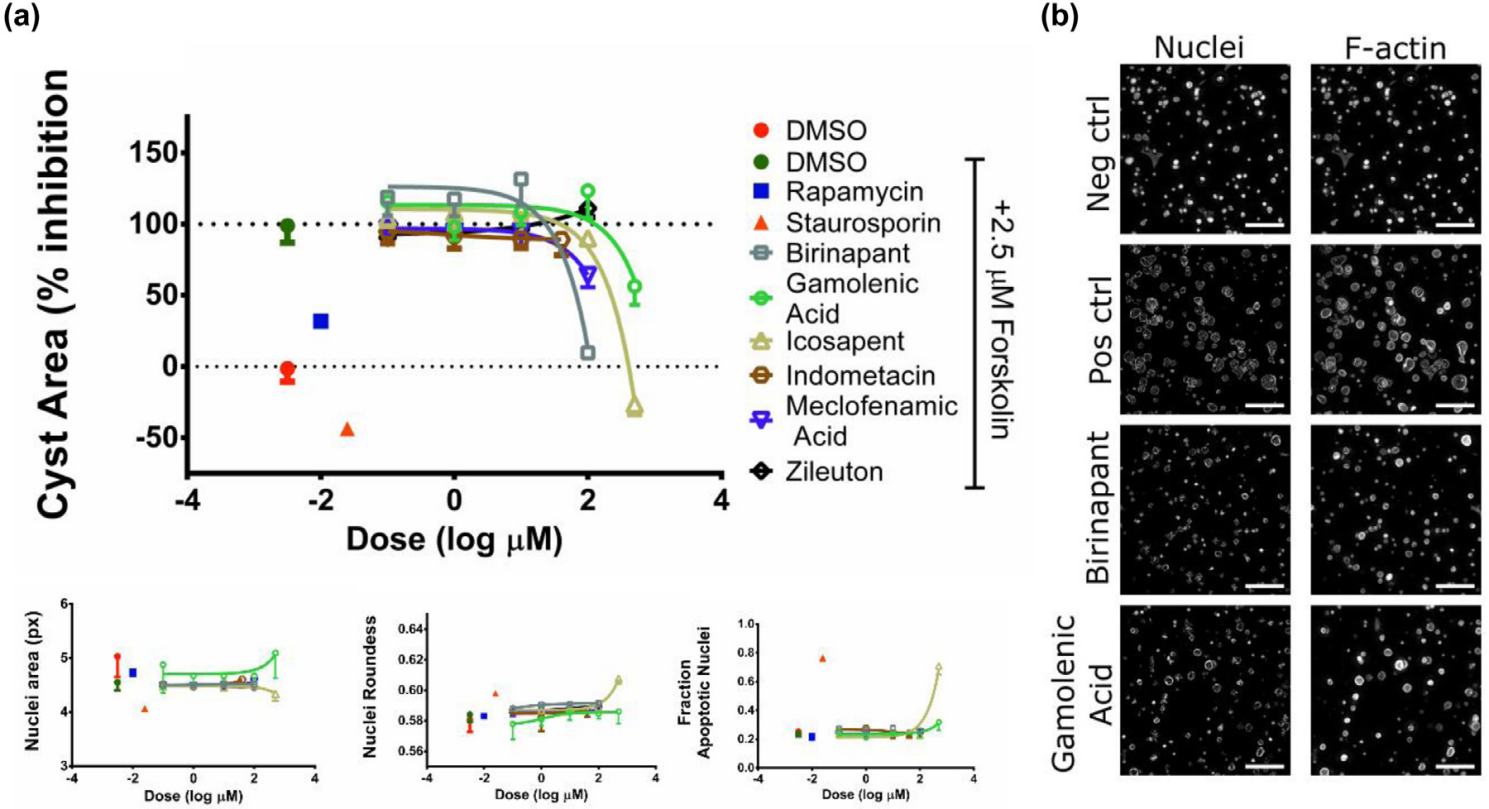
3D-cysts assay of candidate compounds. (a) Top: Quantification of cyst size of the tested compounds normalized to forskolin induced swelling. Reference compounds rapamycin (0.01 µM) and staurosporin (0.25 µM) reduce cyst size, as well as brinapant, Gamolenic Acid, icosapent and Meclofenamic Acid at highest tested concentration of 100 µM, 500 µM, 500 µM and 100 µM respectively (*N* = 4 wells). Bottom: Assessment of staurosporin-like induction of toxicity. Graphs representing average nuclei area, nuclei roundness and the fraction of nuclei that are apoptotic show changes for reference compound staurosporin and for icosapent. (b)Representative images of positive and negative control and two of the test compounds at highest tested dose; 100 µM for brinapant and 500 µM for Gamolenic Acid. Each scalebar is 400 μM.

To identify candidate drugs that might have a favorable effect on ADPKD, we screened the ChEMBL database for drug-protein interactions. From ChEMBL, we only used high quality drug-protein target interactions (See Methods). This generated a list of 990 protein targets (directly assigned targets), and 356,396 interactions with 240,433 compounds. We compared these drug targets to our set of differentially expressed genes. From the total set of 1162 genes that were involved in ADPKD progression, 168 genes were annotated in ChEMBL as candidate drug targets and had enough high-quality bioactivity information to be used in our subsequent analysis (Fig. 4A). These 168 genes were targeted by 48,050 small molecules (Supplementary Table 4: Step 11).

As we were interested in the set of candidate drugs that can be repurposed for ADPKD, we extracted compounds that were tested in 2nd, 3rd or 4th phase clinical trials. 544 out of the 48,050 compounds met these criteria, and these compounds interacted with 111 of our selected targets (Supplementary Table 4: Step 12). Further restriction of the targets by including only those for which the mRNA levels were normalized by treatment with one or more of the three different drugs that slowed cyst formation in our preclinical models. This resulted in a set of 63 unique candidate drug targets interacting with 339 compounds (candidate drugs) (Supplementary Table 5A). For each candidate drug we have obtained its classification in the Anatomical Therapeutic Chemical (ATC) Classification System (https://www.who.int/medicines/regulation/medicines-safety/toolkit_atc/en/) and removed drugs with antineoplastic classification, because they may show too serious side effects during long-term treatment for PKD. 32 unique targets and 116 candidate drugs passed this filtering (Supplementary Table 5B). For each remaining drug-target interaction we looked for information on the mode of action (MoA) of the drug. However, this information was only available for a small subset of these interactions (12 targets and 23 drugs), and we filtered out the targets that had a conflicting direction of dysregulation in relation to the its drug MoA (3 targets were filtered out). We arrived at 29 genes that could serve as a target for drug repurposing in ADPKD (Table 2; Supplementary Table 5B).

**Table 2:** The 29 drug prioritized targets grouped based on their gene category according to MsigDB (*Supplementary Table 5B*).

Gene category	Count of genes	Genes
Protein kinases	3	CDK1
		PRKCB
		PRKCZ
Transcription factors	3	PPARD
		STAT3
		THRA
Cytokines and growth factors/receptors	2	CCL2
		CCR2
Other	21	SLC1A1
		PTGER3
		AKR1B10
		LGALS3
		BIRC2
		HSD17B2
		P2RX7
		PLD2
		CYP2J2
		MGLL
		FKBP4
		PTGES
		ALOX5AP
		P2RY6
		AKR1A1
		MAPT
		CYP51A1
		TRAP1
		AKR1C1
		AKR1C3
		AKR1C2

### 3.7 Selection of candidate drugs

Analyzing the remaining 29 targets, we identified several that were previously linked to ADPKD treatment. For example, Suramin Hexasodium a non-specific inhibitor of P2 receptors inhibiting P2Y and P2X receptors reduced cyst growth in a 3D cysts models while a P2 × 7 receptor antagonist as well as gene knock-down were previously shown to inhibit cystogenesis in a zebrafish model for polycystic kidney disease [36,37]. Suramin is also an antagonist of IL-6, known to inhibit renal fibrosis in chronic kidney disease in rats [38]. Another identified drug, the PPARγ agonist Rosiglitazone, was shown to be effective in animal models for PKD [39], [40], [41]. Pioglitazone, a close PPARγ agonist to Rosiglitazone, is currently undergoing clinical trials for ADPKD [ClinicalTrials.gov Identifier: NCT02697617]. Another prioritized candidate drug was the second mitochondrial-derived activator of caspases (SMAC)-mimetic GT13072, which slowed down PKD progression in two *Pkd1* mouse models [42]. Icosapent also known as ethyl eicosapentaenoic acid, another candidate drug prioritized by our analysis, was shown to reduce PKD severity in a mouse model, but this could not be confirmed in a small clinical trial [43]. Additionally, three candidate drugs, the spleen tyrosine kinase inhibitors entospletinib and R-406, and the polo-like kinase 1 inhibitor BI-2536 were previously shown to be effective in a 3D Cyst screen of the Selleckchem library of compounds [34]. Collectively, these previous findings support our approach in successfully identifying lead compounds for ADPKD drug repurposing.

To identify new candidate drugs for the ADPKD drug development pipeline, we evaluated six compounds in a 3D Cyst assay similar to that performed by Booij et al. [34]. The six candidate drugs, Zileuton, Indometacin, Meclofenamic Acid, Gamolenic Acid, icosapent and Birinapant (Table 3) were selected based on additional evidence for the potential therapeutic potential for ADPKD present in the Euretos Knowledge Platform (https://www.euretos.com/) and the scientific literature.

**Table 3:** Drugs selected for validation in 3D Cyst experiment and their results.

PubChem CID	Drug name	Targets (pChEMBL value^a^)	Results in 3D cyst assay	ATC code (Level 4)
49,836,020	Birinapant	BIRC2 (7.3)	Effective	n/a
5,280,933	Gamma-Linolenic acid (Gamolenic Acid)	PPARD (6.1)	Effective	D11AX
446,284	Eicosapentaenoic acid (Icosapent)	PPARD (5.4)	Effective	n/a
4037	Meclofenamic Acid	AKR1C3 (6.3), AKR1C1 (5.5), AKR1C2 (5.1)	Effective	M01AG
60,490	Zileuton	ALOX5AP (5.5)	Not effective	n/a
3715	Indometacin	AKR1C3 (6.2) , PTGES (4.4) , AKR1C2 (4.3)	Not effective	C01EB
				M01AB
				M02AA
				S01BC

a pChEMBL is a combination of a number of roughly comparable measures of half-maximal response concentration/potency/affinity to be compared on a negative logarithmic scale: -Log(molar IC50, XC50, EC50, AC50, Ki, Kd or Potency). We have tested compounds at 100 μM (pCHEMBL value 4). We have selected targets for which the affinity was a pCHEMBL value > 4.0.

### 3.8 Wet-lab validation of selected candidate drugs

To test the selected drug candidates, we grew renal epithelial cells (mIMRFNPKD 5E4) in a 3D-gel matrix to allow cyst formation. After 96 h, cysts were co-exposed to forskolin, to induce cyst swelling, and the selected compounds for a period of 72 h. Rapamycin, shown before to reduce cyst swelling in several models [34,44], was used as a positive control for cyst swelling inhibition and demonstrated the expected reduction in cyst size (Fig. 4A). Of the selected drug candidates, Meclofenamic Acid, Gamolenic Acid, icosapent and Birinapant slowed cyst growth at the highest concentration tested; 100 µM, 500 µM, 500 µM and 100 µM respectively. Zileuton and indomethacin, however were not as effective (Fig. 4A, top), showing no effect on cyst size on any of the tested concentration up to 100 µM and 40 µM respectively. Birinapant was the most potent compound, with 50% inhibition of cyst swelling around 50 µM. These results were validated in an independent experiment (Supplementary Figure 4). To be able to distinguish true swelling inhibiting properties from severe toxicity, which also leads to reduced cyst size, staurosporin was included as a prototypic toxic compound at. Looking at the effect of staurosporin at 0.25 µM on phenotypic parameters such as nucleus size and shape as well as nucleus fractionation, there is clear induction of cytotoxicity. Of the selected compounds however, only icosapent shows similar kind of phenotypic changes, starting at a concentration of around 100 µM (Fig. 4A, bottom). Representative images of treatment effect can be found in Fig. 4B. These results indicate that 3 out of 6 novel compounds selected through our approach demonstrated to be able to inhibit cyst swelling in vitro without apparent toxicity.

## 4 Discussion

In this study we combined comprehensive gene expression profiling and bioinformatics, with cheminformatics to identify drugs for repurposing and targets to further explore for ADPKD treatment. Our approach is based on an innovative strategy that combines transcriptomics sequencing of different disease states of ADPKD and drug assays databases to arrive to a list of candidate drugs that could have a treatment potential for PKD. Our methodology zooms-in on a set of genes involved in ADPKD progression and proposes candidate drugs that could alter disease progression by targeting relevant genes. Our work is of high relevance to PKD patients since they have limited treatment options. Tolvaptan (Jinarc), the only treatment now available, has limited efficacy, and side-effects like massive diuresis may limit patient adherence [20,45]. Therefore, there is a need for drugs that specifically target the formation and growth of cysts to slow down or halt disease progression. Given the complexity of altered signaling in cyst-lining epithelia, a broad range of potential targets are available, and drug-repurposing is a relative fast strategy for the development of new treatments.

We used a tamoxifen-induced *Pkd1cko* mouse model to generate expression profiles of the kidneys of 7 groups of mice with varying levels of disease progression. Using clustering techniques, we arrived at groups of genes that show altered expression in mild, moderate and advanced stages of the disease, each characterized by increased or reduced activation of certain pathways and pathogenic processes. In the early stage, the TCA cycle, fatty acid biosynthesis, EGFR signaling and G13 signaling were most significantly altered, indicating altered metabolism, proliferation and cytoskeletal remodeling, confirming previous studies in PKD [15,46]. In the moderate phase, we specifically observed increased MAPK and mTOR signaling, both involved in a broad range of cellular processes including cell proliferation and cell stress-related pathways (MAPK) or cell growth, proliferation, protein translation, autophagy, as well as actin cytoskeleton remodeling and apoptosis (mTOR) [23,47,48]. Additionally, at this stage we observed an up-regulation of cytokines such as IL-5 and IL-3, corresponding to inflammatory infiltrates and an active injury response. Inflammation and associated fibrosis became even more prominent in the advanced phase with increased expression of macrophage markers [49,50]. Furthermore, in the late-phase we see evidence of severe cell damage and tissue injury response with the up-regulation of pathways involved in oxidative stress, DNA damage response, and P53 signaling [29,51].

To arrive to a set of candidate drugs that could be repurposed for ADPKD, we took advantage of ChEMBL, where we identified molecules that target genes of the ADPKD progression profile. The advantage of using ChEMBL is that it is based on primary scientific literature, allowing us to validate the source of the bioactivity when needed. However, it should be noted that a similar approach could be envisioned with PubChem Bioassay or another source of biological activities. To make sure that the drug target relationships are of high quality we followed a series of filtering steps that led to 116 molecules binding to 29 genes. It is known that on average *approved* drugs show activity for 6 protein targets, so our selected molecules cannot be considered more promiscuous than normal in particular given that they have gone through phase 1 clinical trials [52]. Our filtering steps aim to minimize the number of ‘wet-lab’ experiments by focusing on only the most relevant and most confident information from literature. To be able to repurpose approved drugs, we did not only retrieve bioactivity data but also retrieved the primary (mode of action) target of each drug. Hence, we also included associated gene targets for approved drug that do not directly relate to the working mechanism described in the literature. As we included only drugs that are used in phases 2, 3 or 4 clinical trials and then filtered out drugs that have antineoplastic effects, we aimed to optimize our selection of drug repurposing candidates. The rationale being that compounds showing toxicity effects in phase 1 drugs known to kill (tumor) cells are less suitable for chronic administration to ADPKD patients. Out of the 116 candidate drugs that we prioritized for ADPKD treatment, we identified 5 molecules that were previously linked to PKD in 3D cultures and/or preclinical studies. More research is required to decide for further clinical development of these drugs/drug targets. Using a 3D-cyst drug screen assay, we have tested the effect of a further 6 drugs on cyst size at four or five dosages. In all cases the screening concentration we used was higher than the noted pChEMBL value (indicating that more than 50% of the compound was bound to the targets). 4 out of the 6 tested drugs had a positive impact on cyst size (decreased cyst size compared to controls). This became more evident at the high dosage, which might suggest a certain toxic effect on the cyst. We further analyzed the toxicological effects of these drugs and our initial toxicology analysis, revealed toxic effects of only 1 of the tested drugs.

The three remaining effective and nontoxic compounds are Meclofenamic Acid, Gamolenic Acid and Birinapant. From Table 3 it follows that the following targets could be responsible for the observed activity of these three compounds: BIRC2, PPARD, and AKR1C1. BIRC2 is the only known target for Birinapant and is in the identified targets. PPARD is a target for both Gamolenic Acid and Icosapent (and in the list of identified targets). AKR1C1, AKR1C2, and AKR1C3 are all in the list of identified targets and have an affinity for the active Meclofenamic Acid. However, the inactive compound Indometacin also has an affinity for AKR1C2 and AKR1C3, ruling them out as the prime targets for Meclofenamic Acid. Finally, PTGES and ALOX5AP seem not to be relevant targets as the inactive compounds Indometacin and Zileuton have affinity for them. It should be noted that the here retrieved targets represent only the targets for which activity was measured in the scientific literature; absence of these measurements does not demonstrate the absence of potential affinity. Moreover, the tested compounds may also have more targets on which they may demonstrate affinity (Supplementary Table 6). However, we selected in our approach only genes that were shown to be affected in ADPKD, which is not true for the other targets listed in Supplementary Table 6.

For the identified drugs we were also able to obtain more relevant information from literature, interestingly all these results are in line with our findings from Table 3. Meclofenamic Acid has been identified to target aldo-keto reductase family 1, which is implicated in steroid metabolism [53], which was reported to be involved in cyst development in cpk rat, a PKD model [54]. Gamolenic Acid has been selected based on PPARδ, which controls an array of metabolic genes involved in glucose homeostasis and fatty acid synthesis/storage, mobilization and catabolism. For other PPAR family members, PPARα and PPARγ, are being studied in (pre)clinical trials for PKD [40,55]. Birinapant is a SMAC mimetic and known modulator of apoptosis, which binds to and inhibits the activity of Inhibitors of Apoptosis Proteins (IAPs), including BIRC2(=cIAP1) thereby freeing caspases to activate apoptosis [56]. Another SMAC mimetic, GT13072, was previously shown to slow down PKD progression in *Pkd1* mouse models [42]. Overall, these drug candidates are relevant to the molecular events involved in ADPKD progression. However, further testing and pre-clinical experiments are needed to determine the efficacy of these drugs for ADPKD treatment.

To our knowledge this is the first drug repurposing effort in ADPKD at this scale. It expands on the previous transcriptomics efforts performed by others in the field. In this study we used deep RNA-sequencing of ADPKD transcriptomics across multiple disease stages, rather than microarrays [15,29,57,58]. The aforementioned studies differ in several elements, most notably their source of studied samples. Where we and Menezes et al. used adult *Pkd1* mutant mice, Pandey et al. used embryonic kidneys of *Pkd1* mutants and both Song et al. and de Almedia et al. used patient obtained ADPKD kidneys of ADPKD patients. Despite these differences, comparable dysregulated pathways have been reported. In all studies, abnormalities in metabolism, cell cycle and cell death are observed. Our results suggest that irregulates in metabolism and cell growth could play a role in early cyst development. Furthermore, we sequenced drug-induced ADPKD models to target progression involved genes at a higher precision, and thus enabling enhanced drug-repurposing. Our method screens thousands of approved drugs for their potential to treat ADPKD, expanding the work of others that focused on studying a selected number of drugs [59], [60], [61], [62], [63].

Although our approach is supported by wet-lab and *in silico* experiments, we acknowledge several limitations of our study. (1). For the adult onset PKD mice, we only included males, while several results suggest ADPKD presentation differences between males and females [64]. Despite the differences in progression rates, gene network analyses revealed that the underlying mechanisms of PKD progression between male and female mice do not differ [15]; (2) Our starting point was gene expression data, while not all molecular processes act through changes in gene expression. Stage specific proteomics data and analysis of posttranslational modifications would be needed to obtain a more comprehensive insight in the molecular pathways associated with disease progression and would improve the quality of our drug predictions; (3) Drugs and their targets are biased towards the most studied drugs, diseases, and proteins (i.e. enzymes and G protein coupled receptors make up more than 75% of the data), while less-well characterized drugs may constitute equally good candidates for drug repurposing strategies [65]; (4) Further functional wet-lab experiments would be needed to determine the exact contribution of each gene to ADPKD progression and cyst growth. As more data will be implemented in ChEMBL and other biomedical database in the future, the power of this approach will increase. In addition, this approach is widely applicable to other diseases as well, provided that large scale high quality transcriptomic/proteomics data is available to be compared to databases cataloging drug affinity and activity towards a broad range of protein targets.

## Funding sources

The research leading to these results has received funding from the People Program (Marie Curie Actions) of the European Union's Seventh Framework Program FP7/20072013 under Research Executive Agency Grant Agreement 317246 and under grant agreement 305444 ‘RD‐Connect’, and the Dutch Technology Foundation Stichting Technische Wetenschappen Project 11823, which is part of The Netherlands Organization for Scientific Research and a grant from the Dutch Kidney Foundation (17PhD02). The funders didn't have any role in study design, data collection, data analysis, interpretation, writing of the report.

## Author contributions

T.B.M., W.N.L, P.A.C.t.H. and D.J.M.P. conceived and designed research; T.B.M., W.N.L., H.B., Z.G. and G.J.P.V.W. performed experiments; T.B.M., W.N.L., H.B., Z.G., G.J.P.V.W. and K.M.H. analyzed data; T.B.M., W.N.L., L.S.P., P.A.C.t.H. and D.J.M.P. interpreted results of experiments; T.B.M. and H.B. prepared figures; T.B.M. drafted manuscript; T.B.M., W.N.L., H.B. Z.G, K.M.H., G.J.P.V.W., L.S.P. P.A.C.t.H. and D.J.M.P. edited and revised manuscript; T.B.M., W.N.L., Z.G., H.B., K.M.H., G.J.P.V.W., L.S.P., P.A.C.t.H. and D.J.M.P. approved final version of manuscript.

## Declaration of Competing Interest

Kristina M. Hettne performed paid consultancy between November 1, 2015 and March 31, 2018 for Euretos B.V, a startup founded in 2012 that develops knowledge management and discovery services for the life sciences, with the Euretos Knowledge Platform as a marketed product. Leo Price is a founder shareholder and Hester Bange employee at OcellO B.V., which operates in the PKD drug discovery field. All other authors have nothing to disclose.

## Appendix Supplementary materials

Image, application 1

Image, application 2

Image, application 3

Image, application 4

Image, application 5

Image, application 6

Image, application 7
