# Supplementary material for: Prioritization of novel ADPKD drug candidates from disease-stage specific gene expression profiles
Source: eBioMedicine. 2019 Dec 24;51:102585. doi: 10.1016/j.ebiom.2019.11.046 (PMC7000333; doi:10.1016/j.ebiom.2019.11.046)
Supplement: Supplementary file 1 [file mmc1.docx]

**Supplementary Figures**

**Supplementary Figure-1:** A schematic representation of the overall methodology used in deriving the results


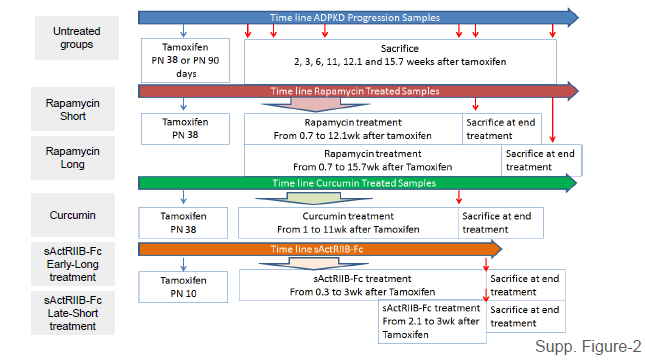


**Supplementary Figure-2:** The different mouse groups used in creating the ADPKD progression profile and mouse groups with drug treatments. The ADPKD progression profile was created using untreated groups, that contains *Pkd1del* tamoxifen induced at either PN38 or PN90 and sacrificed at different time points to create different levels of disease progression. Drug treatment was applied on three groups; Rapamycin and Curcumin treatment were given to *Pkd1del* tamoxifen induced at PN38, and sActRIIB-Fc treatment was given to *Pkd1del* tamoxifen induced mice at PN10.

**Supplementary Figure-3:** Correlation between 2KW/BW% and Cystic Index (%), as measures of disease progression. To ensure that 2KW/BW% reflects PKD disease severity of the mouse models used in this study, we plotted the cystic indices of individual mice to their corresponding 2KW/BW% in four different experiments. PKD was induced by tamoxifen administration at P38 in the short-term Rapamycin (A) and Long-term Rapamycin (B) experiments (23). PKD was induced by tamoxifen administration at P40 in the Curcumin (C) experiment (26). PKD was induced by tamoxifen administration at P10 in the Activin (D) experiment (24). Linear regression was applied through all data points of the different graphs and showed significant correlation between 2KW/BW% and Cystic Index of all experiments (23, 24, 26). The P-values (t-statistics) for the slope in the linear regression and the squared Pearson correlation are shown.

**Supplementary Figure-4:** Quantification of cyst size of the tested compounds normalized to forskolin induced swelling. Reference compounds rapamycin (0.01µM) and staurosporin (0.25µM) reduce cyst size, as well as brinapant, Gamolenic Acid, icosapent and Meclofenamic Acid at highest tested concentration of 100µM, 500µM, 500µM and 100µM respectively (N = 4).
