# Supplementary material for: Prioritization of novel ADPKD drug candidates from disease-stage specific gene expression profiles
Source: eBioMedicine. 2019 Dec 24;51:102585. doi: 10.1016/j.ebiom.2019.11.046 (PMC7000333; doi:10.1016/j.ebiom.2019.11.046)
Supplement: Supplementary file 2 [file mmc2.docx]

**Supplementary Table 6:** All activities in ChEMBL release 22 for drugs selected for validation in 3D Cyst experiment and the experimental result (in bold identified targets in ADPKD profile)

| **Drug Name** | **Target(s) from**  **ChEMBL 22**  **(pChEMBL value^1^)** | **Results in 3D**  **Cyst assay** | **ATC Code**  **(level 4)** |
| --- | --- | --- | --- |
| Birinapant | **BIRC2 : 7.3** | Effective | n/a |
| Gamolenic acid  (Gamma-Linolenic acid) | PPARA : 6.6  **PPARD : 6.1**  PPARG : 5.7  SRD5A1 : 4.9  F3 : 4.5  L3MBTL1 : 4.5 | Effective | D11AX |
| Icosapent  (Eicosapentaenoic acid) | PPARA : 6.0  PPARG : 5.8  OXER1 : 5.7  **PPARD : 5.4**  ALDH1A1 : 4.4  KMT2A : 4.3  CYP19A1 : 4.3  F3 : 4.1 | Effective | n/a |

| Indometacin | AR  9.9  IL8 : 7.3  COX1 : 6.9  GPR44 : 6.3  **AKR1C3 : 6.2**  COX2 : 6.0  NPSR : 5.8  SLC22A6 : 5.5  mTOR : 5.3  SLC22A8 : 5.2  RAB9A : 5.2  ALOX5 : 5.2  HIF-1 : 5.1  KDM4D : 5.0  LMNA : 5.0  SLCO1B1: 4.9  ABCC1 : 4.9  DHRS9 : 4.9  CYP2D6 : 4.8  MAPT : 4.7  GLO1 : 4.6  CYP2C9 : 4.6  ABCB1 : 4.5  SLCO1B1: 4.4  **PTGES : 4.4**  PPARG : 4.3  **AKR1C2 : 4.3**  AKR1C4 : 4.3  PMP22 : 4.1 | Not effective | C01EB  M01AB  M02AA  S01BC |
| --- | --- | --- | --- |
| Meclofenamic Acid | COX2 : 7.1  COX1 :6.7  **AKR1C3 : 6.3**  MAPK1 : 5.5  **AKR1C1 : 5.5**  TTR : 5.3  **AKR1C2 : 5.1** | Effective | M01AG |
| Zileuton | LMNA : 6.7  LTB4R : 6.4  EPHX2 : 6.2  ALOX5 : 6.0  **ALOX5AP : 5.5**  GAA : 4.9 | Not effective | n/a |
